# Development of a model for predicting hospital beds shortage and optimal policies using system dynamics approach

**DOI:** 10.1186/s12913-022-08936-w

**Published:** 2022-12-14

**Authors:** Seyede Maryam Najibi, Seyed Hosein Seyedi, Payam Farhadi, Erfan Kharazmi, Payam Shojaei, Sajad Delavari, Farhad Lotfi, Zahra kavosi

**Affiliations:** 1 Health Human Resources Research Center, School of Management and Medical Information Sciences, Shiraz University of Medical Sciences, Shiraz, Iran; 2 Department of Industrial Engineering and Management, Shahrood University of Technology, Shahrood, Iran; 3 Department of Management, Faculty of Management and Accounting, Zand Institute of Higher Education, Shiraz, Iran; 4 Department of Management, Faculty of Economic, Management and Social Science, Shiraz University, Shiraz, Iran

**Keywords:** System dynamic, Hospital beds, Policy, Simulation, Modeling

## Abstract

**Background:**

Policymakers use simulation-based models to improve system feedback and model the reality of the problems in the system. This study uses the system dynamics approach to provide a model for predicting hospital bed shortages and determine the optimal policy in Shiraz, Southern Iran.

**Methods:**

This study was designed based on Sterman's system dynamic modeling (SDM) process. Firstly, we determined the main variables affecting bed distribution using a mixed qualitative and quantitative study which includes scoping review, expert panel, Delphi, and DANP. Then, dynamic hypotheses were designed. Subsequently, we held several expert panels for designing the causal and stock-flow models, formulating and testing a simulation model, as well as developing various scenarios and policies.

**Results:**

Dynamic modeling process resulted in four scenarios. All of the scenarios predicted a shortage of national hospital beds over a 20-year time horizon. Then, four policies were developed based on the changes in the number of beds and capacity of home care services; finally, the optimal policy was determined.

**Conclusions:**

Due to the high cost of setting up hospital beds, developing and supporting cost-effective home care services, strengthening the insurance coverage of these services, and improving the quantity and quality of community care, considering the real needs of the community could be considered as an optimal option for the future of the city.

**Supplementary Information:**

The online version contains supplementary material available at 10.1186/s12913-022-08936-w.

## Introduction

Provision of high-quality medical services is one of the core responsibilities of governments. All people, regardless of their socio-economic status, should have equitable access to healthcare [1]. Health system reform is widely considered in many countries to ensure equitable access to universal healthcare [2].

In some developing countries, the allocation of health system resources does not reflect the real needs of society due to a lack of solid evidence for decisions [3]. The number of hospital beds, as one of the most important sources of medical services, is the primary indicator of estimating the capacity of a region in providing medical services. Accordingly, the number of hospital beds required in an area is the basis for the estimation and allocation of human resources, medical equipment, and support facilities [3, 4].

According to the World Bank, in 2017, the highest bed-to-population ratios were related to Japan, South Korea, and Bulgaria, with 13.05, 12.43, and 7.45 hospital beds per 1000 population, respectively. Simultaneously, Afghanistan, Nigeria, and Guatemala had the lowest bed-to-population ratio, representing limited health resources in low-income countries. According to the World Bank, the ratio in Iran was 1.6 in 2017, which was far from the global average of 2.89 [5].

The scarcity of resources and unlimited health demands, create unique economic situations for hospitals [6]. Inappropriate distribution and shortage of hospital beds lead to several consequences, including a decrease in staff-to-patient ratio, limited capacity of service delivery, decreased patient safety and satisfaction, reduced quality of care, increased risk of medical errors and infections, increased risk of job burnout due to workload, and finally poor distribution of nurses, physicians, and medical equipment [7, 8].

Management of hospital beds more effectively requires prediction of how these resources should be distributed. The allocation of resources in the health system is often based on previous allocations and is rarely prioritized based on the real needs of the regions. Also, this issue is often influenced by political interests [9, 10]. However, decisions about the development and distribution of hospital beds have different dimensions, including the number of patients, hospital indicators such as bed occupancy rate, number of physicians, length of stay, age and sex structure of the population, and population growth. These factors affect the demand for hospital beds and constantly change the behavior of the health system [11–14].

The effect of conditions such as population growth, population transition, increasing expectations, increasing need for more resources, and the imbalance between supply and demand for resources requires policymakers to have an effective tool to determine the causes of any imbalance. This is achieved by monitoring the interrelationships of various components of the health system, analyzing the impact of exogenous factors, determining the causes of existing problems, and evaluating the effects of policies [15]. As a result, policymakers need to use more systematic methods to learn about the behavior of social systems such as the healthcare system to cope with the growing uncertainty and increasing complexity of the systems [16]. Healthcare organizations show complexity in both detailed and dynamic parts and consist of many components and interrelated subsystems. Because of the necessity of allocating resources through more effective and evidence-informed decisions, health policymakers are encouraged to use simulation-based models to respond better to the complexity of the system [17]. In this regard, Davahli et al. (2020) investigated the application of system dynamics simulation in healthcare through a systematic review. This review provides a comprehensive picture of the applications of system dynamics methodology to address complex healthcare issues. The results of this review showed that since 2013 the application of systems dynamics has attracted the attention of many health researchers in patient flow, obesity, labor demand, and HIV/AIDS [18].

Hospital beds are costly to build and maintain, but a shortage of beds in an emergency can lead to increased mortality. Therefore, the planning of the number of hospital beds available over time should be sufficiently accurate. Because of the effect of various factors on the number of hospital beds, uncertainty about the optimal amount of these resources, long-term changes such as variations in patients’ number due to immigration or population growth, as well as interrelationships of variables affecting the need for hospital beds, it is necessary to investigate the need for hospital beds, taking into account cause-effect relationships through a system dynamics approach. This approach, with a dynamic view on dealing with time, paying attention to uncertainty in parameters, taking into account the relationships between variables, examining time delays, and above all, showing the feedback effects of variables over time, seeks to understand and predict the behavior of the model variables in the considered system [19]. Therefore, the present study recommends an optimal policy using the system dynamics approach based on predicting hospital beds in Shiraz city over a 20-year time horizon.

## Methods

### Study design

This research was designed based on Sterman’s system dynamic modeling (SDM) process. SDM consists of six steps, which include identifying the key variable, creating the causal model, designing the stock-flow model, formulating the simulation model, testing the model, and developing various scenarios [20]. In recent decades, the SDM has been used as a management tool to understand real-world behavior and implement strategic policies. According to Sterman (2002), SDM is a perspective and a collection of conceptual tools that allows us to understand the structure and dynamics of a complex system [21]. This method is an approach used for detecting the nonlinear dynamic behavior of a system and investigating how the structure and parameters of the system lead to behavioral patterns. Another main purpose is to develop effective and rigorous policies to improve the management system performance. The application of this method can also be a great help in predicting the results of policies in order to optimize decisions [22]. Application of SDM has increased dramatically in the last ten years and has been used in the field of healthcare since the 1970s on population health issues [23].

The main purpose of this study was to predict the shortage of hospital beds and recommend an optimal policy based on the dynamics of systems. In this regard, a case study has been conducted in public hospitals affiliated with Shiraz University of Medical Sciences, Shiraz, Iran. In the first step of the study, a mixed quantitative and qualitative study was done to find out the key variables affecting the distribution of hospital beds in a region. The following steps were taken to model the distribution of hospital beds and develop policies for the future of bed distribution in Shiraz. This study was approved by the ethics committee of Shiraz University of Medical Sciences with the code IR.SUMS.REC.1399.340.

### Identification of the key variables

To avoid an unnecessary increases in model complexity in SDM, it is of utmost importance to understand the main variables and exclude those that do not play a key role in problem-solving [24]. In this regard, the most important factors that affect the distribution of hospital beds in a region were retrieved through a mixed qualitative and quantitative study which included scoping review, expert panel, Delphi, and DANP. The methodology and results of the first phase of our research are published in a separate report [25]. Accordingly, the variables with the most weight and importance were selected to incorporate into the causal model. These variables included population, population growth rate, patient-to-population ratio, total number of patients, number of specialist doctors, bed occupancy rate, number of travelers to the specialist, the average length of stay, and capacity of home care services. In addition, some variables were used in the model due to the need to formulate the model and establish mathematical relationships.

### Creation of the causal model

The basic notion of SDM is to understand how objects interact in a system. The causal model in SDM is an abstract and conceptual model that offers the interaction hypotheses of selected variables [21, 26]. The causal loop diagram (CLD) is a tool used in this model to depict the feedback structure of the systems and the relationships between variables in the problem situation. The elements inside the diagram are the key variables that have the most significant impact on the system performance and are interconnected by arrows that indicate causal effects. Each connection is given a pole (" + " or "-") that shows the influence of the independent variable on the dependent one. These poles explain the systems structure [27, 28]. There are two types of feedback loops in the model. Reinforce loops cause the system to spiral out of control, whereas balancing loops keep it balanced [15].

In this study, to design a causal model of hospital bed distribution, six experts in the field of health services management and health policy, as well as three operational research specialists attended four expert panels. We applied purposeful sampling to invite the experts who were expected to have the highest degree of knowledge about the issue. One of the main purposes of using this method is collective thinking among a group of experts or stakeholders on a set of topics to provide in-depth insights and policy recommendations [29].

All relevant ethical considerations were observed during the panels, and the participants were first informed about the study objectives and completed an informed consent form. The participants were also assured about the confidentiality of the information and the anonymous transfer of the results. As a result, causal correlations and loop were established after four two-hour sessions, and the causal model was designed in a reciprocal process.

### Designing the stock-flow model

**Fig. 1:**
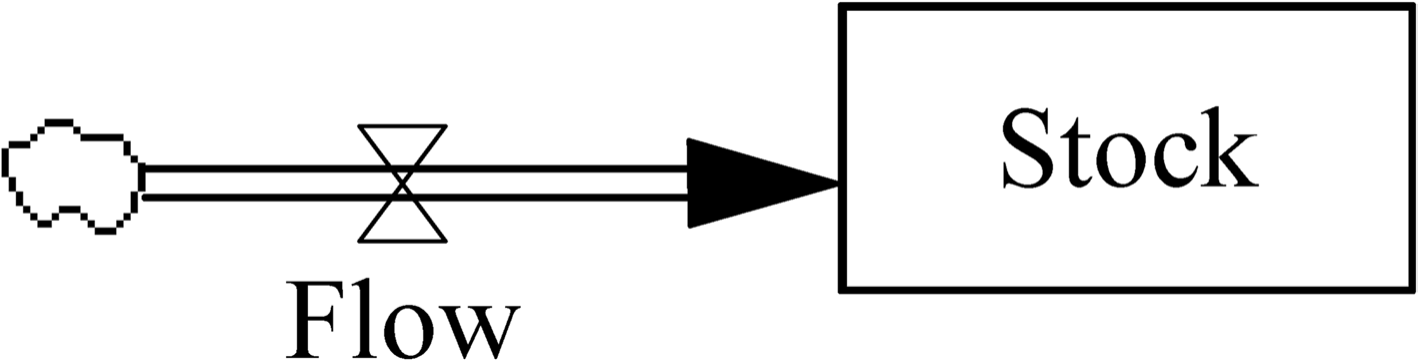
Simple stock and flow diagram

The basis of SDM is the acquisition and representation of the feedback process, which defines the dynamics of the system through the structure of stock and flow variables, time delay, and nonlinear functions [30]. Stock-flow models are ideal for quantifying cause-and-effect relationships. As Fig. 1 shows, this model has numerous fundamental components. The stock variable representing the accumulation of resources and its current state depends on what happened in the previous time period. The stock variable is indicated by a rectangle. The flow variable reflects the change of the stock variable and is visually represented by a symbol that looks like a faucet on a pipe. The external source is shown as a "cloud", indicating that analysis of that source is not appropriate for this model. This model also includes parameters with constant values. The model may also contain "auxiliary" or "soft" factors that may not have a direct effect on the system but affect its behavior [31].

In this step, the experts participated in three two-hour sessions to identify the variables and evaluate their connections based on the causal model. Finally, the stock-flow model was considered as a diagram that helped to write equations.

### Data entry and formulation of the simulation model

For creating the mathematical equations for the model, five two-hour meetings were held in the presence of a group of operational research specialists. The model variables were determined and entered into the PLE version of VENSIM software. Data were gathered from 2015 to 2019 to analyze the present trend and calibrate the model. The data required to run the model were collected from various sources, including the statistical yearbook of Iran and Fars province, the annual performance report of the Ministry of Health, the AVAB operational software (hospital statistics and information system of the Ministry of Health), and the health information technology unit in public hospitals. Finally, simulations were run for this model with a 20-year time horizon using VENSIM software, and the necessary analysis was completed. The time horizon considered for this study was20 years, from2015 to 2034. Since actual data of the model variables was available up to 2019, to measure the validity of the model, we compared the simulation results from 2015 to 2019 with the real data, after which the simulation continued up to 2034 for 15 years. The basis for determining the time horizon mentioned above was the experts’ opinion and the general rule in system dynamics studies. The time horizon must extend far enough into the future to observe the rate and speed of changes. Moreover, to obtain the important dynamics and the feedbacks which generates them, we had better consider the time horizon several times longer than the time delays of the system and even a little longer [21]. The simulation results on the behavior of key variables were the basis for analyzing the scenarios and policies in the following steps.

### Model testing, design of scenarios and policies

The structural validity of the causal and stock-flow models in terms of variables and the relationships between them was confirmed based on expert opinion. To ensure that the behavior of the model demonstrates the actual conditions, we validated the behavioral validity of the model through marginal limit tests, compatibility of numerical functions, as well as equilibrium state and conformity with actual behavioral patterns.

In this step, five two-hour sessions were held with experts to determine the scenarios and policies. To develop the scenarios, we considered the macro-trends as the current events that are causing problems nationwide. At this stage, scenario analysis should be done to predict future events, so that researchers consider a wide variety of issues, trends, and decision-making points that are likely to be met in the future [32].

## Results

Using the ANP method, variables with higher weights were determined to enter the causal model. Selected variables included the capacity of home care services, population, number of specialist physicians, patient-to-population ratio, the average length of stay, bed-to-population ratio, population growth rate, bed occupancy rate, and the number of people who travel to receive medical services.

**Fig. 2:**
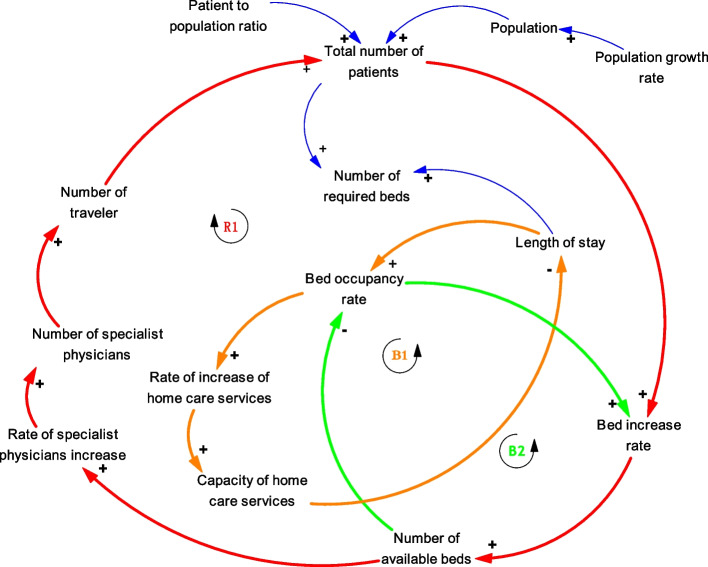
Causal loop diagram of hospital beds distribution

The hospital bed distribution model is a conceptual model that includes selected elements and their interaction hypotheses. The dynamics of the model are determined by the feedback loops in the CLD. Figure 2 depicts the CLD. The variables of patient-to-population ratio, population growth rate, and population are considered exogenous in this model.

In the reinforcing loop R1, as the number of patients grows, the rate of growth of hospital beds increases which leads to a rise in the number of available beds. Following the increase in the number of beds, the rate and number of specialist physicians will increase. Then, the number of people who travel to receive medical services increases, and this leads to an increase in the total number of patients.

In the balancing loop B1, an increase in bed occupancy rate in hospital and completing their capacity can motivate home care providers, thus leading to a rise in the growth of home care services. By referring patients to home care centers, the length of stay in hospitals is reduced, and consequently, the bed occupancy rate is reduced.

In the balancing loop B2, when hospital bed occupancy rates rise, the bed increase rate rises to suit the patients’ demand; hence, the number of available beds increases. As a result, the bed occupancy rate decreases (Fig. 2).

**Fig. 3:**
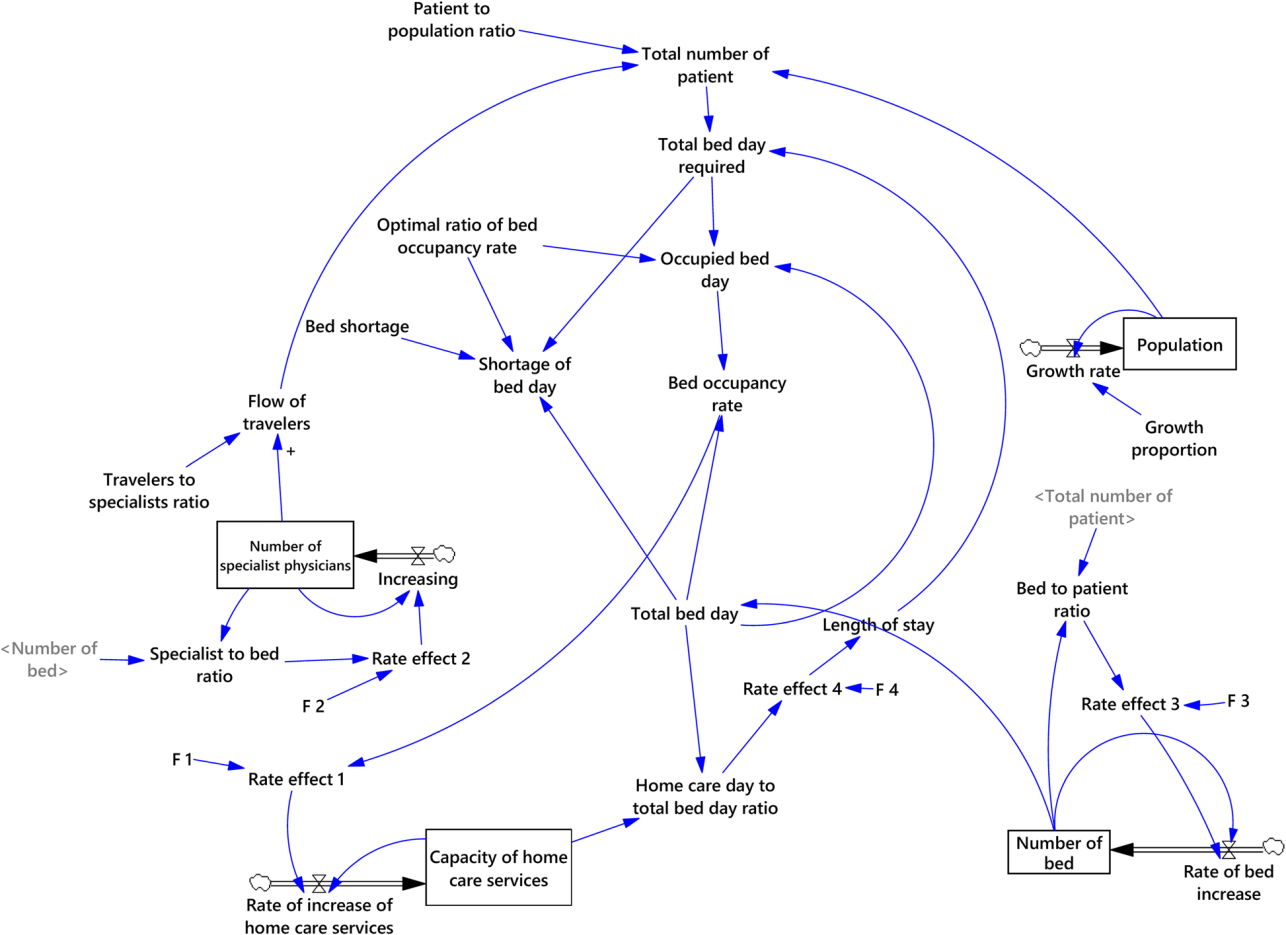
Stock-flow model of hospital beds distribution

After determining the key elements and formulating the causal model, the elements should be defined quantitatively, and their effects should be formulated mathematically. The stock-flow model for the distribution of hospital beds is shown in Fig. 3. Population, number of beds, number of specialist physicians, and capacity of home care services were selected as stock variables in this model. The other variables were determined as flow and auxiliary variables.

### The main elements in the stock-flow model

Population growth can affect the total number of patients. Patient-to-population ratio as a parameter can also affect the total number of patients. The total number of patients might affect the total bed days required, bed occupancy day, and consequently, bed occupancy rates. In this model, the optimal ratio of bed occupancy rate was considered 85%, and a variable called bed shortage was incorporated into the model to predict the bed shortage rate for the coming years as a main variable in the scenarios.

On the other hand, the total number of patients may influence the bed-to-patient ratio which, through a rate effect (Rate effect 3), influences the rate of bed increase. The rate effect in the model is a variable that is defined through a Lookup Table or function table (F 3). This table comprises two axes, x, and y, which in this part of the model, (x) represents the bed-to-patient ratio and (y) shows the rate of bed increase. The values for the lookup table are determined according to the literature review, global standards, and expert opinion.

On the other side of the model, the number of beds affects the specialists-to-bed ratio through a rate effect (Rate effect 2) and can influence the rate of specialist increase. The rate effect 2 is determined by lookup table (F 2), in which (x) is the specialists-to-bed ratio and (y) is the rate of specialist increase. The ratio of travelers to specialists is considered a parameter that can affect the flow of travelers and thus changes the total number of patients.

The bed occupancy rate can affect the rate of increase in home care services and, consequently, the capacity of home care services through a rate effect (Rate effect 1) and a lookup table (F 1). Due to the impact of home care services on the length of stay in the hospital, the capacity of home care services affects the length of stay through the variable of home care day to total bed-days ratio and a rate effect (Rate effect 4).

Finally, for the stock variables, the data for 2019 were presented as initial values, and the data for flow variables in the range of 2015 to 2019 were treated as historical data. Consequently, a 20-year time horizon was considered for the simulation (Fig. 3).

The data used in the model and the associated calculations are presented in Additional file 1. Moreover, Table 1 shows the formulas determined for the variables in the model.

**Table 1:** Formulas of the stock-flow model for the distribution of hospital beds

1	Number of bed: 3560
2	Growth proportion: 0/014
3	Population: 1,557,600
4	Growth rate: Growth proportion * Population
5	Total patient: Flow of travelers + Population * Patient to population ratio
6	Patient to population ratio: 0/129
7	Total bed day required: Total patient * Length of stay
8	Bed occupancy day:IF THEN ELSE (Total bed day required ≤ Total bed day * Optimal ratio bed occupancy rate, Total bed day required, Total bed day * Optimal ratio bed occupancy rate)
9	Bed occupancy rate: Bed occupancy day / Total bed day
10	Total bed day: Number of bed * 365
11	Shortage bed day: MAX(0, Total bed day required-Optimal ratio bed occupancy rate * Total bed day)
12	Capacity of home care services: 76,650
13	Specialist to bed ratio: Number of specialist physicians / Number of bed
14	Number of specialist physicians: 690
15	Flow of travelers: Number of specialist physicians * Travelers to specialists’ ratio
16	Rate effect 1: F 1 (Bed occupancy rate)
17	Rate effect 2: F 2 (Specialist to bed ratio)
18	Rate effect 3: F 3 (Bed to patient ratio)
19	Rate effect 4: F 4 (Home care day to total bed day ratio)

### Suggested scenarios and policies

**Fig. 4:**
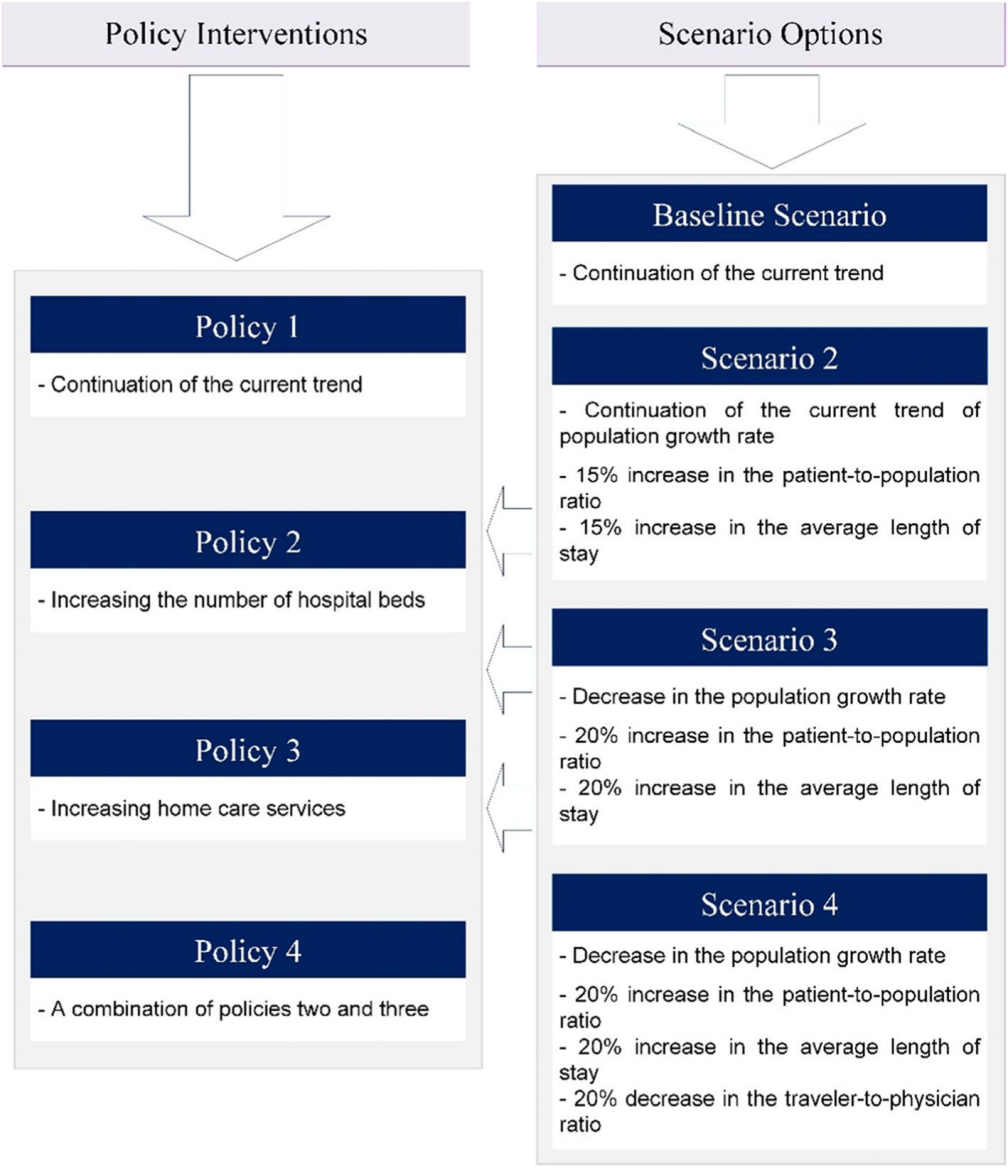
Scenario options and policy interventions

As to designing the scenarios and policies in this study, it is emphasized that the three main factors that make up scenarios are the variables which are subject to environmental changes, including population, patient-to-population ratio, and travelers-to-specialists ratio. Moreover, two factors that can be changed by policymakers and develop our policies are the increase in beds and home care services. Therefore, with this approach, four scenarios and four policies were developed in this study (Fig. 4).

In the baseline scenario, all variables are changing as before. In the following scenarios, two assumptions have been considered, which are the reduction of the population growth rate due to the fertility situation in Iranian society and the continuation of the population growth rate as before. In addition, due to the increasing trend of population aging in Iran, the ratio of patients-to-population is increasing, and also following the aging of these patients and exposure to chronic diseases, the length of stay is also considered to increase.

Another hypothesis of the study was that with a more equitable distribution of physicians in the province, the number of travelers seeking medical services in Shiraz city would decrease.

The findings revealed that by running the model in the baseline scenario, the most important result was facing the shortage of hospital beds from the seventh year. In the second scenario, the shortage of hospital beds started from the third year. In the third scenario, the shortage of hospital beds began after about two years and two months, and in the fourth scenario, it started in the third year.

In the assumed scenarios, to solve the problem of bed shortage, we developed four policies for each scenario and a total of 16 situations. In all situations, policy number one depicts the baseline status, and no particular intervention is made.

**Fig. 5:**
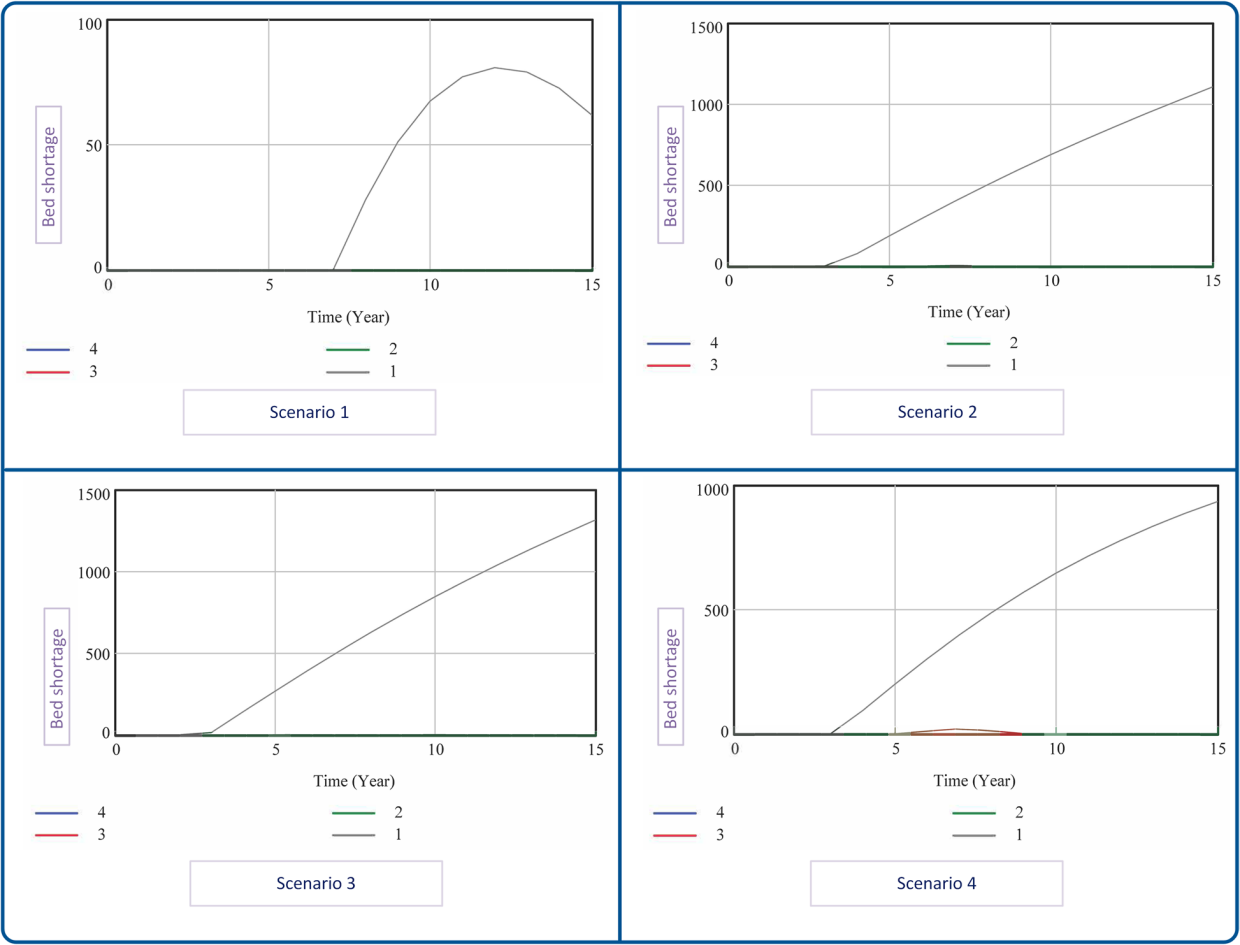
Shortage of hospital beds in run scenarios & policy

In the second policy, only the number of beds and in the third policy, only the number of home care services increased to such an extent that the shortage of beds reached zero in the coming years. In the fourth policy, the number of beds and number of home care services were increased simultaneously to solve the problem (Fig. 5).

The results suggested that policy number four, compared to policy number two which solely considered bed increases, was the best-case scenario, as the simultaneous increase in home care and the number of beds in all study scenarios eliminates the need to increase approximately 300 beds (Table 2).

**Table 2:** Number of hospital beds and home care services in the end simulation

Scenario	Policy	Number of Bed at the End Simulation	Capacity of Home Care Services at the End Simulation
**Scenario 1**	P1	4872	115,110
	P2	5141	113,913
	P3	4872	133,509
	P4	4979	126,330
**Scenario 2**	P1	4935	116,811
	P2	6892	112,737
	P3	4935	288,407
	P4	6570	228,985
**Scenario 3**	P1	4942	117,076
	P2	7296	112,327
	P3	4942	332,689
	P4	7007	258,444
**Scenario 4**	P1	4897	116,930
	P2	6518	113,893
	P3	4897	282,182
	P4	6263	226,674

## Discussion

In healthcare systems, many internal and external factors affect healthcare outcomes, which are often nonlinear. Under these conditions, simulation models can help elucidate the non-intuitive behavior of complicated healthcare problems. SDM have been used to investigate the influence of various policies on the behavior of any complex system, taking into account all levels of the system, handling all events that occur throughout the system lifetime, enhancing system feedback, and modeling the reality of system issues [28, 33].

In this study, three causal loops were designed based on expert opinion and the literature. In the reinforcing loop R1, based on the study of Ordu et al. (2020), the number of patients can affect the number of beds [13]. With the increase in hospital beds, the need for medical specialists to provide medical care to patients is increasing. As the study of Horev et al. (2004) showed, there was a positive correlation between the distribution of physicians and hospital beds [34]. In the continuation of this process, as the number of doctors in an area increased, the flow of travelers to receive medical services in that area increased. For example, a study in New Zealand highlights the role of travelers flow in the distribution of beds and doctors [35].

In the balancing loop B1, adequate incentive is created for the centers that provide home care services by increasing the bed occupancy rate in hospitals and completing their capacity. In this regard, the study of Gordon et al. (2018) also underlines the existence of home care centers and their role in reducing the burden on hospitals and more effective allocation of beds and staff in hospitals. Consequently, it also affects the reduction in the patients’ length of stay [36].

In the balancing loop B2, as bed occupancy increases and hospital bed capacity completes, the number of beds should be increased to meet patient needs, which can help reduce the occupancy rate of the beds. The study of Green (2002) shows that both at the macro-policy level and the managerial level of health centers, the bed occupancy rate of current hospitals has been considered the most important variable for determining the need for beds [37].

The simulation results for the distribution of hospital beds show that if, according to the baseline scenario of this study, all variables continue as before, the shortage of hospital beds will start from the seventh year of the simulation. However, the variables will change over time, which can be embedded in SDM studies in possible scenarios. Among the macro trends in the country, demographic trends are the issues used in many social and economic studies in education, healthcare, the labor market, and long-term services (such as pension policies, urban development, and social welfare) [38]. The population is one of the most important factors that should be considered in planning the capacity of hospital beds. In this area, the world is facing the phenomenon of population aging. In developing countries and Asian countries, however, the aging process is much faster than in Western countries. According to the Asian population aging report, the aging of the population occurs faster than the economic growth [39, 40]. In Iran, statistical indicators show that population aging has begun due to increased life expectancy and reduced fertility rates [41]. According to international statistics, 21.7% of Iranian population in 2050 will be over 60 years old [42].

Changes in the age of the population and the transition to aging in Iran can have a significant impact on changing the pattern of illnesses to chronic diseases and future demands for care and hospital beds (patient admission rate) as well as provision of services (average length of stay) [43]. Therefore, in this study, possible scenarios for population growth rate, increase in patient-to-population ratio due to population aging, and increase in the length of stay due to chronic disease in the elderly were designed. By implementing the scenarios, the simulation results showed a shortage of beds from almost the third year. Population aging will be accompanied by an increase in the number of required beds. Studies by Schofield et al. [44] and Ian et al. [45] show that in Australia, with the incremental increase in the number of patients aged 75 and over, the number of hospital beds required by the year 2050 will increase by about 62 percent.

According to the scenarios developed in this research, to address the problem of shortage of hospital beds, four policies were considered based on changing the number of beds and home care services. The results showed that in terms of practical and economic considerations, policy number four is the best option because increasing the number of hospital beds alone will be costly, so it is estimated that the cost of setting up a hospital bed in Iran is more than forty thousand dollars, equivalent to 10 billion Iranian Rials.

Many successful health systems worldwide have adopted policies to reduce hospital dependency and bed-based care to move the patients out of the hospital and provide community care and alternative services such as home care and long-term care centers (LTC) [46]. According to the study of McKee et al. (2004), the number of hospital beds needed in a country depends on the availability of alternative care facilities [47]. In addition, to decrease dependence on hospital beds, authorities should take measures to reduce inappropriate admissions, increase the efficiency of hospital care, and facilitate faster discharge, which often requires the development of alternative facilities and services such as home care [47]. The NHS in the United Kingdom, for example, avoids hospitalization for mental healthcare and instead provides services through community-based multidisciplinary teams while people live in their own homes [48]. These services are delivered in people's homes, nursing homes, and LTC facilities [49]. Increase in life expectancy and the high burden of chronic illness have made the expansion of LTC a high-priority policy in many countries [50]. According to Chen et al. (2010), the elderly refer to public hospitals regardless of their illness and use the same treatment methods as other patients. Complex procedures might cause delays in diagnosis and treatment and increase the costs [51]. In a 2015 study, Rashwan et al. (2015) investigated a solution to overcome the problem of delayed discharge of elderly patients and plan to meet the growing demand for hospital beds in Ireland over the next five years. This study examined the impact of different policies on hospital bed capacity over the next five years. These measures include a 20% increase in the elderly care places, a 15% reduction in the admission of the elderly in acute care beds, and the effect of home care packages on increasing discharge from long-term care centers [52]. In this regard, Imison et al. (2017) and Edwards et al. (2014) found that although there is differing evidence regarding the cost savings of relocating healthcare, strengthening out-of-hospital care is justified because early intervention and support can help people avoid costly hospital care while providing peace of mind in the family setting [53, 54].

### Limitations of the study

Considering the Covid-19 pandemic and its impact on hospital indicators such as bed occupancy rate, the average length of stay, patient admission rate, as well as more use of home care services in the early months of the pandemic, data for this period were not entered into the simulation model to take the ordinary conditions of the health system into account. Another limitation of this study was the omission of some indicators affecting the distribution of beds due to the lack of available information. According to the main purpose of the study, the researchers did not conduct a particular survey on the cost of setting up a hospital bed compared to the cost of home care services in the year of the study. Therefore, it is suggested that in future research, the costs and, if necessary, the amount of economic savings in the hospital system should be investigated.

In future studies, it is necessary to convert some variables that influence hospital indicators, such as technological progress and e-health, into quantitative variables through Fuzzy methods and insert them into the model. Although this study provided evidence for hospital bed allocation policies, the economic analysis of the proposed policies remains an area for further research. Future research is also recommended to explore the role of health information management and emerging technologies such as telemedicine, mobile health, and e-health in reducing reliance on hospital beds.

## Conclusions

Hospital bed allocation is an important and complex process involving many factors. Using a systems approach and a system dynamics model, the shortage of hospital beds in Shiraz was predicted with a time horizon of 20 years, and policies aimed at avoiding shortage of beds were proposed.

Based on the findings of this research, developing and supporting cost-effective home care services, strengthening the insurance coverage of these services, and improving the quantity and quality of social services following the real needs of the community could be considered an optimal option for the future of the city. According to the general framework and structure of the model, the model designed in this study can be applied to other cities and provinces using their specific data.

## Supplementary Information

**Additional file 1.**

## Data Availability

All data generated or analyzed during this study are included in this published article and its supplementary information files.

## Abbreviations

SDM: System dynamic modeling
CLD: Casual loop diagram
DANP: Dematel analytic network process
F: Function
P: Policy
